# Interpersonal communication as an agent of normative influence: a mixed method study among the urban poor in India

**DOI:** 10.1186/s12978-015-0061-4

**Published:** 2015-08-12

**Authors:** Rajiv N. Rimal, Pooja Sripad, Ilene S. Speizer, Lisa M. Calhoun

**Affiliations:** Department of Prevention and Community Health, George Washington University, Washington, D.C., USA; Department of International Health, Johns Hopkins University, Baltimore, MD USA; Department of Maternal and Child Health, University of North Carolina at Chapel Hill, Chapel Hill, NC USA; Carolina Population Center, University of North Carolina at Chapel Hill, Chapel Hill, NC USA

**Keywords:** Social norms, Interpersonal communication, Contraceptive use, Urban India

## Abstract

**Background:**

Although social norms are thought to play an important role in couples’ reproductive decisions, only limited theoretical or empirical guidance exists on how the underlying process works. Using the theory of normative social behavior (TNSB), through a mixed-method design, we investigated the role played by injunctive norms and interpersonal discussion in the relationship between descriptive norms and use of modern contraceptive methods among the urban poor in India.

**Methods:**

Data from a household survey (*N* = 11,811) were used to test the underlying theoretical propositions, and focus group interviews among men and women were then conducted to obtain more in-depth knowledge about decision-making processes related to modern contraceptive use.

**Results:**

Spousal influence and interpersonal communication emerged as key factors in decision-making, waning in the later years of marriage, and they also moderated the influence of descriptive norms on behaviors. Norms around contraceptive use, which varied by parity, are rapidly changing with the country’s urbanization and increased access to health information.

**Conclusion:**

Open interpersonal discussion, community norms, and perspectives are integral in enabling women and couples to use modern family planning to meet their current fertility desires and warrant sensitivity in the design of family planning policy and programs.

## Background

Interventions seeking to change fertility desires and contraceptive practices are important in countries such as India, where early childbearing rates are high and maternal morbidity and mortality are common [1]. According to the National Family Health Survey 3, the state of Uttar Pradesh—the site for the current study—has both the highest fertility rate and one of the lowest rates of contraceptive use in India. The state’s total fertility rate (TFR) has declined from 4.06 children per women in 1999 to 3.82 in 2006 (national TFR = 2.7), and the proportion of women using any modern method of contraception increased from 20.8 percent in 1999 to 29.3 percent in 2006. The state, however, lags behind the national average of 48.5 percent [2]. Use of modern family planning methods in Uttar Pradesh is particularly low among the less wealthy [3], socially disenfranchised [2], and parents with only girls (as opposed to boys) [2]. Lower education is also significantly associated with lower use of modern contraception [3, 4]. Living in urban poor environments further limits access to health services and is associated with high unmet family planning needs [5–7].

A number of social and cultural factors at the familial and community levels present barriers to women’s use of modern family planning [1, 4, 8]. One important barrier is the influence of community norms on women’s decisions to seek care in reproductive health clinics [9]. In the broader literature, social norms are considered important determinants of health behaviors [10–12], and there has recently been a marked increase in norms-based studies across various health domains [13]. In the literature on family planning practices, scholars are also recognizing the importance of considering the role of social norms at both the individual [8, 14] and community levels [15]. Important questions still remain. It is not known, for example, what role interpersonal communication plays in normative influences, even though norms are understood as fundamentally social processes [16–18]. Similarly, different types of social norms have been shown to influence behaviors in varying ways [11, 19, 20], and hence there is a need for greater specificity in hypotheses related to normative influences. This study seeks to fill this gap in knowledge.

### Theory of normative social behavior

The theory of normative social behavior (TNSB) [21] posits that both descriptive norms (people’s perceptions about the prevalence of a behavior) and injunctive norms (the extent to which people perceive social pressures to conform) affect behaviors [11]. It further predicts that the influence of descriptive norms on behaviors is moderated by a number of factors, one of which is injunctive norms. Put another way, individuals’ propensity to enact a behavior is governed by their perceptions of others’ behaviors (descriptive norms), social sanctions for failure to conform (injunctive norms), and the interaction between these two factors, such that the highest compliance occurs when people believe that they will incur punishments if they do not engage in a popular behavior. This particular hypothesis has been tested in a number of health domains, including alcohol consumption [22, 23], conservation behavior [24, 25], physical activity [26], and voting behavior [27]. To date, this has not been tested on contraceptive use. Our first hypothesis tests this proposition.

Furthermore, even though norms are social phenomena propagated through communication, the extent to which interpersonal communication modifies the relationship between descriptive norms and behaviors has also not received adequate attention in the literature. In this paper, we seek to understand the underlying process and also test the proposition that the interaction between descriptive norms and interpersonal communication will be such that higher levels of contraceptive use will be observed among those who perceive higher levels of use among their peers and amongst those who engage in higher levels of discussion about contraceptive use.

## Methods

Adopting a sequential mixed-method design, we first test our hypotheses and then explore, through qualitative data, the extent to which inter-spousal communication and normative considerations manifest in decisions about contraceptive use. For ease of presentation, we refer to the quantitative study as Study 1 and the qualitative study as Study 2. After summarizing the primary findings from each study in the Results section, we integrate the findings from both studies in the Discussion section.

### Data sources

Data for this study come from four cities (Agra, Aligarh, Allahabad, and Gorakhpur) in Uttar Pradesh, India. Data were collected in 2010 at baseline, before the start of an intervention, called the Urban Health Initiative (UHI), designed to increase access to and use of modern contraceptives among the urban poor. This was part of a larger initiative (the Urban Reproductive Health Initiative-URHI) that runs interventions simultaneously in Kenya, Nigeria, and Senegal. Ethics approval was obtained from both in-country and the appropriate United States-based university institutional review boards.^1^

### Study 1

Our hypotheses in Study 1 were two-fold. First, we proposed that the association between descriptive norms and modern contraceptive use would be greater when injunctive norms were strong (as compared to when they were weak). Second, we also proposed that the association between descriptive norms and modern contraceptive use would be greater when interpersonal communication was high (as compared to when it was low).

This study utilized a unique approach for mapping and identifying slum residents to oversample the urban poor (many of whom live in unregistered areas). Lists of officially registered slums, spatial imagery, and ground reconnaissance were used to identify slum areas within each study city. These slum areas were divided into areas of approximately 100 structures to be used as primary sampling units (PSU). Areas of the city not identified as slums were also divided into areas of approximately 100 structures, to serve as non-slum PSUs. Slum and non-slum PSUs were numbered, and a representative sample of 64 slum- and 64 non-slum PSUs were selected in each city. Slum PSUs were intentionally oversampled in order to permit domain specific analyses by slum or non-slum. Descriptive statistics were weighted using cross-city weights, but given similar results from the weighted and unweighted multivariate analyses, for simplicity, we report the unweighted multivariate results in this paper.

Household listing and mapping activities were carried out in all selected PSUs to ascertain household eligibility. A random sample of 30 households was selected from the list of eligible households in each PSU. In selected households, all currently married women age 15–49 were eligible for study participation. Eligible women were approached for interview and asked for verbal consent for participation. For women between 15 and 17 years of age, a guardian provided consent and the eligible woman provided assent. Upon consenting, women were asked questions about reproduction, contraception, fertility preferences, discussion about family planning, gender inequality, and media exposure. In total, 11,811 currently married women across the four sites provided information on contraceptive use (weighted number is 12,794 women across the four cities).

#### Measures

- *Contraceptive Use.* The dependent variable, modern contraceptive use, was measured by asking participants if they or their spouse were currently doing something or using any method to delay or avoid getting pregnant. If the respondent answered affirmatively, she was then asked what type of contraceptive method she (or her spouse) was currently using. Based on the responses, participants were classified as modern users or non-modern users (includes traditional method users and non-users). Hence, the dependent variable was coded dichotomously—non-user or user of traditional methods (coded as 0) and modern method user (coded as 1).
- *Parity Groups.* We sought to determine whether the impact of norms differed for women with no children, women with only one child, and women with two or more children. Consequently, this parity variable was stratified into three categories for analysis.
- *Descriptive Norms.* Descriptive norms were elicited by asking women about their perceptions of how many couples in their area used family planning. Responses were *none* (coded 1), *some* (2), *most* (3), or *all* (4). Approximately 30 % of women reported “don’t know” to this question. We examined them in more depth and found they were not different demographically from those who provided a response, and thus we imputed the mean values pertaining to the parity group to which they belonged.
- *Injunctive Norms.* Injunctive norms focused on women’s perceptions about their husbands’ support for contraceptive use. Women were asked whether they believed their husbands approved of couples using modern contraceptive methods to space births or avoid pregnancy. Their responses were coded dichotomously for approval (1) and disapproval (0); those who responded “don’t know” (less than 2 % of the sample) were classified as 0.
- *Interpersonal Communication.* Women were asked whether they ever discussed family planning with their spouse, specific family members, friends, and neighbors. Responses (each coded dichotomously as 0 or 1) were added into an index (α = 0.61, mean = 2.22, range = 1–8).
- *Control Variables.* Control variables included age, education, and wealth. In order to assess non-linear trends, age in years was squared and used in the model as a predictor. Education level was measured as a categorical variable, ranging from “none” to “12+ years” (see Table 1 for categories). Wealth indices were computed based on 27 available household assets reported as part of the household survey. Principal components analysis was undertaken and a factor score was developed for the first factor. The household sample was divided into weighted quintiles (groups of 20 % each) across all cities based on the factor score. The individual-level wealth categorization was based on the respondent’s household-level quintile. Religion was classified as Hindu, Muslim, or Other, and it was modeled as a binary variable in the regression models, with Hindu as the referent group. Given that fewer than one percent of the sample fell into the “other religion” category, these respondents were combined with those self-reporting as Muslim for analytic purposes. City was classified as Agra, Aligarh, Allahabad, and Gorakhpur and modeled as binaries with Agra as the referent group.

**Table 1:** Descriptive statistics for weighted sample of women across study cities in Uttar Pradesh, India, 2010

	Women with no children (*n*_*w*_ = 1126) % or mean (SD)	Women with 1 child only (*n*_*w*_ = 2003) % or mean (SD)	Women with 2 or more children (*n*_*w*_ = 9271) % or mean (SD)	*p*-value (Chi-squared or *t*-test)^a^
Age, *M* (*SD*)	23.8 (5.7)	26.5 (6.2)	34.9 (7.3)	<.001
Education (%)				<.001
None	19.82	16.76	36.67	
<5 years	2.16	1.97	2.93	
5–7 years	9.44	7.16	9.66	
8–9 years	13.17	9.97	9.99	
10–11 years	12.21	12.05	11.13	
12+ years	43.2	52.1	29.55	
Wealth (%)				<.001
Quintile 1	15.21	12.21	19.67	
Quintile 2	21.29	14.84	18.86	
Quintile 3	18.32	18.72	20.23	
Quintile 4	22.61	25.17	21.03	
Quintile 5	22.57	29.07	20.22	
Religion (%)				>.05
Hindu	81.69	79.78	79.49	
Muslim & Other	18.32	20.07	20.51	
City (%)				<0.001
Agra	31.66	34.87	33.80	
Aligarh	17.11	12.93	17.75	
Allahabad	26.38	30.12	26.78	
Gorakhpur	24.85	22.08	21.67	
Descriptive norms, *M* (*SD*)^b^	1.83 (0.59)	1.82 (0.58)	1.82 (0.54)	>.05
Injunctive norms (%)	93.56	96.82	95.50	<.001
Interpersonal communication, *M* (*SD*)^c^	1.92 (0.74)	2.19 (0.73)	2.26 (0.67)	<.001
Modern contraceptive use (%)	4.81	35.69	53.27	<.001

^a^Compares differences across the three parity groups. ^b^Perception that others in the community use family planning (4-point scale). ^c^Interpersonal communication around family planning (8-point scale). Unweighted samples: *n* = 1134, *n* = 1749, *n* = 8928

Logistic regression was used to assess main-effects and interaction effects. Interaction effects were tested by including the cross-product in the regression model that included the two corresponding main-effects (between descriptive norms and injunctive norms and between descriptive norms and interpersonal communication).

### Study 2

The purpose of the qualitative study was two-fold. First, we explored the nature of descriptive and injunctive norms and the role of interpersonal communication on family planning. Second, given the importance of husbands’ attitudes and beliefs uncovered in Study 1, we included the perspectives of both women and men to better understand the emergence of family planning norms within their particular social environments.

Data for the qualitative study come from same-sex focus group discussions (FGDs) conducted among women and men, segmented by parity, in the same four cities as noted in Study 1. FGDs were selected as the appropriate method because of their utility in obtaining group attitudes or norms on a topic.

The data collection team, in collaboration with members of non-governmental organizations who had extensive experience working in their respective cities, first identified catchment areas for recruitment. From these areas, households were randomly selected, and one person from each household who met the inclusion criteria (adult age, appropriate gender to meet the quota, and parity) was chosen (at random if more than one eligible adult resided in the home). The selected adult was asked to come at a particular time to a previously identified location for the focus group discussion. Across the four cities, 36 focus groups (16 with women and 20 with men) covered three parity groups: those without children (*n* = 13), with only one child (*n* = 12), and with two or more children (*n* = 11). A moderator and one note-taker facilitated each FGD, and topics included beliefs, attitudes and knowledge about fertility, family size and structure, and family planning practices and consequences. Socio-cultural pressures, norms, and stigma associated with each of these domains were probed during each session. All discussions were recorded, transcribed, and subsequently translated into English. The translated text was coded inductively in Atlas.ti, and ambiguities in meaning were resolved by consulting project staff.

## Results and interpretation

### Study 1

The study population in the three parity groups—women with no children (weighted *n* = 1126), women with one child only (*n* = 2003), and women with two or more children (*n* = 9271)—is shown in Table 1. The socio-demographic characteristics, norms, and behaviors related to uptakeof modern contraceptives varied to some extent among the groups. Not surprisingly, women at higher parity were older. Compared to the other two groups, women with two or more children were significantly more likely to have had no education. The distribution of wealth also varied significantly across the three groups, with single-parity women having the greatest wealth. The religious distributions of women were fairly consistent across the three groups, with Hindus making up the majority, followed by Muslims (about a quarter in all groups). Cross-city distributions across parity groups indicate that the Agra sample of women was largest, followed by Allahabad, Gorakhpur and Aligarh, respectively.

Zero-order correlations were computed across all variables to assess overlapping variance. Table 2 shows that all demographic variables, interpersonal communication, and norms-related variables were significantly associated with use of modern contraceptives. This table also shows that, besides a high correlation between age and parity, as expected, the remaining correlations were small and there was little problem with multicollinearity for the multivariate analyses.

**Table 2:** Zero-order Pearson Correlations (*N* = 11,794)

	(2)	(3)	(4)	(5)	(6)	(7)	(8)	(9)	(10)
1. Age	−.08***	.12***	−.02*	.02*	.50***	.16***	.01	−.01	.03**
2. Education	1.00	.61***	−.12***	.11***	−.19***	.08***	.07***	.10***	.01
3. Wealth		1.00	−.03***	−.03***	−.07***	.11***	.06***	.11***	.01
4. Religion			1.00	−.05***	.01	−.08***	−.06***	−.07***	−.04***
5. City				1.00	−.02**	.01	.00	−.02*	−.18***
6. No. children					1.00	.29***	.01	.04***	.13***
7. Contraceptive use						1.00	.04***	.12***	.15***
8. Descriptive norm							1.00	.02	−.01
9. Injunctive Norm								1.00	.09***
10. Interpersonal communication									1.00

*p < .05, **p < .01, ***p < .001

Logistic regressions showed that, adjusting for covariates, higher descriptive norms were associated with an increase in contraceptive use only in the highest parity group (see Table 3). Women with two or more children who perceived a higher number of women in their community using contraception were themselves more likely to use contraceptives. Likewise, among women in the highest parity group, injunctive norms were significantly associated with contraceptive use; this relationship was only marginally significant (*p* < 0.10) among women with single parity, and it was not significant among women without children. Interpersonal communication was associated with increased contraceptive use among all three parity groups. Thus, normative influences (descriptive and injunctive) appear to come into play once women have had more than one child, but interpersonal communication about family planning may be important for all groups as a potential motivator of contraceptive use.

**Table 3:** Multivariate logistic regressions (Odds Ratios), by parity, assessing modern contraceptive use, urban Uttar Pradesh, India

	No children (*n* = 1123)	1 child only (*n* = 1745)	2 or more children (*n* = 8921)
Control variables			
Age	1.19	1.17*	1.56***
Education	1.44**	1.24***	1.03*
Wealth	1.17	1.26***	1.13***
Religion (Ref = Hindu)	0.92	1.10	0.69***
Aligarh (Ref = Agra)	1.38	0.73****	0.65***
Allahabad (Ref = Agra)	1.12	0.76****	1.02
Gorakhpur (Ref = Agra)	0.41*	0.80	1.05
Descriptive norms (DN)	0.86	1.15	1.09*
Injunctive norms (IN)	1.72	2.11****	2.57***
Interpersonal communication (IPC)	1.38****	1.28***	1.36***
DN × IN^a^	0.48	0.12*	0.95
DN × IPC^a^	2.39**	2.37***	1.45***

*p <0.05, **p <0.01, ***p <0.001, ****p <0.1

^a^With the exception of interaction estimates, all coefficients are included in the main effects model

**Fig. 1:**
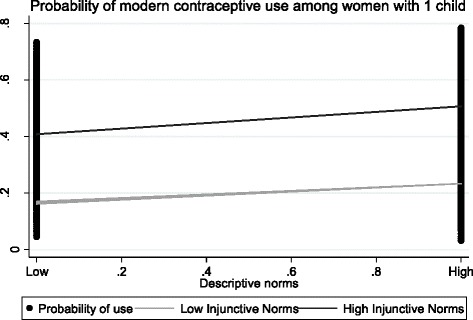
Interaction between descriptive norms and injunctive norms (women with one child). Interactions are adjusted for all covariates

**Fig. 2:**
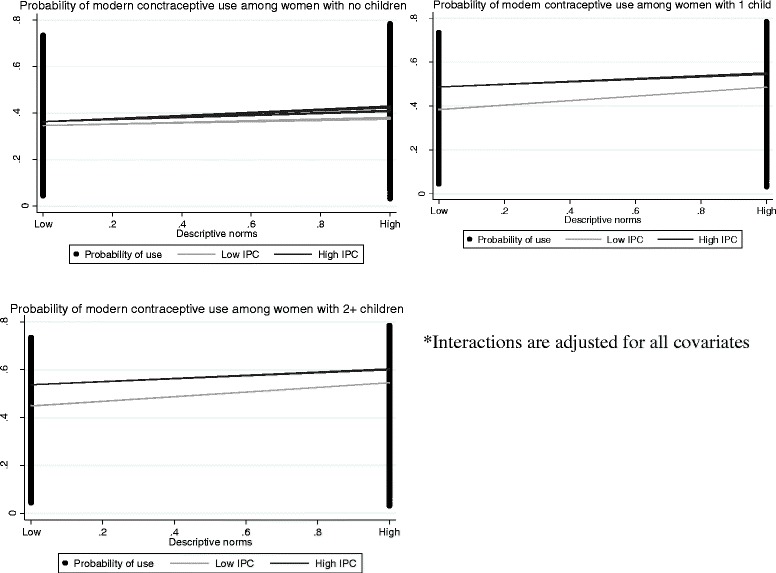
Interactions between descriptive norms and interpersonal communication (all three parity groups)

The descriptive norm and injunctive norm interaction term was significant only for single-parity women. The descriptive norm and interpersonal communication interaction term was significant across all three parity groups (Table 3). Further analysis of interaction patterns showed that among single parity women, the association between descriptive norms and contraceptive use was stronger when injunctive norms were strong than when injunctive norms were weak (Fig. 1). Similarly, the association between descriptive norms and modern contraceptive use was greater when interpersonal communication was high than when interpersonal communication was low. These patterns of interactions are shown in Fig. 2.

### Study 2

The open-ended nature and breadth of topics covered in the discussions allow for this study to limit its scope to parts of the transcripts that specifically focus on norms. Through inductive and deliberative analytic processes, we identified descriptive and injunctive norms around fertility and family planning decision-making, overall, and at different parity stages. Norms around childbearing were particularly strong across respondents in influencing contraceptive use. Beyond the theory of normative social behavior, experientially-driven norms (i.e. perceptions of family planning) emerged as an area to further explore in urban Uttar Pradesh.

#### Childbearing norms

Participants expressed a strong desire to have children relatively quickly after marriage, and they also believed that others in their social environment (including parents and community members) shared this norm. This childbearing-timing norm, which arose repeatedly in our data, across parity groups, operated both as a descriptive and an injunctive norm. Embodied in the sentiment of “incompleteness” of a home without children, a woman from Agra expressed her view about the community-shared feeling of loneliness that leads to the urgency felt by childless couples.

*“There is nothing without kids. It is because of children that there is happiness in the house. We don’t have kids, so we are trying to do so.”* (Female, Agra, no children)

This sentiment was often mediated through an injunctive norm—of feeling pressured or incurring community judgment for not having children, as suggested by the quotes below. Participants articulated the social demand for couples to have children to prove their worthiness, particularly for women, and dispel any myths or perceptions of “bad spirits” that may underlie a couple’s lack of progeny.

*- “And if you can’t conceive in one or two years after marriage, people make bad comments and abuse you.”* (Female, Allahabad, no children)

*- “Everyone starts commenting about her and calling her sterile.”* (Female, Gorakhpur, one child)

To avoid stigmatization, couples often had at least one child quickly after marriage. The data suggested that childbearing was a social obligation, which, if not met, had consequences that extended beyond the intergenerational contract. It portended future suffering in old age because children would not be there to look after them.

*“In a family, one child is necessary. If any troublesome circumstances arise for parents in the future then the child will be a supportive pillar for them…..As compared to males [sons], females [daughters] are more supportive. Daughters take care of their parents, provide them support and assume responsibility for their well-being.”* (Male, Agra, no children)

The sentiment described in the above quote suggests unique intergenerational contracts for daughters and sons, while also challenging the well-recognized son preference and traditional roles played by sons in India [28]. The relative consistency of reporting equal sex preference across FGDs may be explained by the fact that our sample was urban. Since prior studies focus on rural populations or report averages (rural majority), it may be that urban poor, because of their distinctive goals and needs, succumb less to the pointed desire for sons. This qualitative finding is in contrast with quantitative data from the same cities; prior analysis suggests that the son preference remains and is associated with family planning use [29]. The same study also demonstrates that while there is a preference for sons, there is also a desire in urban Uttar Pradesh for family compositions that include both sons and daughters—our qualitative data are congruent with this finding.

Both men and women expressed aspirations to limit family size to two to three children. This preference was repeatedly attributed to increasing expenses related to care-taking and the need to provide adequate food, shelter, education, and opportunities to succeed. One male in the highest parity group (Agra) spoke of how, in larger families, happiness and closeness decrease, saying that “*If families are smaller, there is more affection.*”

#### Decision-making norms around family planning

Decisions about family planning choices were reported by all groups as being shared among women, their spouses, and families. The descriptive norm emerged that males often assumed a dominant position in terms of decision-making power when it came to issues around family planning and the adoption of a contraceptive method. From the male perspective, though discussions with spouses were important, the implicit understanding revealed that the final decision rests with the husband.

In joint families, parents or parents-in-law often have high levels of decision-making power. This authority stems in part from the norms of reciprocity that involve elders providing support for their children’s family, and vice versa.

Injunctive norms surrounding son preference were strong, and wives often felt pressured to give birth to a boy, a factor that determined the use of contraception. This is in line with prior quantitative analyses done in urban Uttar Pradesh [29]. Overall, however, both men and women felt that couples should jointly, through discussion, make decisions on family planning. One of the male participants without children from Allahabad provided an illustrative analogy of the importance of shared decision-making: *“It is necessary because husband and wife are the tires of a car…if there is no talk between them, then how will the car proceed?”* We explore this idea further in the next section on the nuances of the interpersonal communication between spouses and others.

#### Interpersonal communication: substance and context

Interpersonal communication appeared to play a critical role in couples’ decisions to use contraceptives and in their normative understanding (descriptive and injunctive) about benefits and consequences of using contraceptives. Women and men tended to report joint decisions between spouses around family planning and contraception. Embedded in these decisions were high levels of inter-spousal communication and a deliberative process that often took place in private. Specifically, both women and men reported speaking at night, “in the bedroom,” or when they were together “out in the evening,” in the absence of family members such as elders or children.

Responses to questions about the nature of these conversations showed thematic congruence with the desire to have a small family and to share each other’s true opinions (“*she tells of her heart and I tell her of mine*”—Male, Aligarh, no children). These conversations suggested a level of interpersonal influence between husbands and wives, with husbands' approval or disapproval taking precedence in affecting decisions about contraceptive use.

Although interview moderators failed to probe for specific reasons for not discussing family planning methods with the spouse, a number of males reported not discussing it with their wives. We infer, based on field notes and consultation with data collectors and moderators, that hesitance in the low parity groups (particularly groups without children) to talk about bearing children and family planning reflected a social awkwardness and a normative “taboo.” One example, from a men’s focus group in Aligarh, was the following: *“No, we [the family] have not talked to her [the wife] about this [FP].”* Informal discussions with researchers and staff involved in the data collection process revealed the difficulty of motivating younger women with no children to engage in discussion. Many of them, newly married, often through family arrangements with little prior familiarity with their husbands’ families, found themselves assimilating to a new environment in which they were reluctant to express personal views openly.

Interpersonal communication also influenced normative effects mentioned previously around childbearing and contraceptive decisions. Patriarchal traditions and the need of the couples to consult with other family members, usually their parents living in the same household, are critical to the family planning decision-making process. Our transcripts suggested that, among women, it was common to discuss family planning with female relatives and friends; however, many of the women considered their husbands’ preferences as being particularly important. Males mentioned talking with male heads of the family (usually their fathers), and women with female heads.

Though deference to elders’ preferences on family planning was a salient theme, this attitude appears to be changing for women, particularly those who have had at least one child. Friends of the same gender were consistently cited as influential as a source of family planning information. This inter-friend dialogue appeared to be a comfortable process for many women, and it has an educational capacity to spread descriptive norms around contraceptive use.

*“When someone who uses it [Copper-T, an intra-uterine device] comes to us and suggests using this [method], we ask about it. I never applied it, but my relative’s daughter-in-law used it. I had not even thought about it…”* (Female, Agra, two or more children)

As the above excerpt suggests, informal discussions between friends and relatives around their positive experiences with using particular contraceptives such as Copper-T, pills, condoms, and others may motivate uptake in a community.

#### Shifting norms and practices

In light of the various normative and interpersonal channels for communication around family planning practices we observed, norms and the role of women in urban Uttar Pradesh appear to be shifting over time. The importance of considering women’s wishes and joint-decision-making, as noted by men, suggests that perhaps some of the traditional power structures are beginning to relax in the context of contraceptive decision-making. When asked about the decision-making process around condom use, one man responded:

*“I will take my wife’s advice. We both need to agree …… her advice is compulsory. If she refuses, then I will have to re-think.”* (Male, Aligarh, no children)

The relatively high level of respect accorded to the wife in decision-making is representative of some of the men across the four cities and in different parity groups. A potential explanation for this phenomenon, emerging from our data, is likely the response to shifting economic norms of more women entering the workforce and assuming greater responsibility. This challenges the previous notion that because males are the primary earners in the family, their decisions related to reproductive health are weighted more heavily. A male in Allahabad reflected: *“Whatever the husband wants happens… because the husband earns.*” When further probed on how he felt the situation would differ if the wife earned an income, he responded: “*Then they both have to think and mix their opinions.”*

In some cases, women exerted greater autonomy to use contraceptives. For example, when husbands and families were non-responsive to a wife’s attempts to initiate conversation about contraception, women, particularly in higher parity groups, sought care themselves. They often opted for a female-centric method because these provided an avenue through which they could limit and space births in a secretive way.

*“If they [husbands] are not ready to listen to you at night, then in the morning you can take pills [emergency contraception]. If husbands are not listening, people say that we should use Copper-T, so we use it.”* (Female, Agra, two or more children)

This type of agency - in contrast to previous studies [30] -  reflected a number of women’s gradual increase in exercising choice in the context of reproductive decision-making. Surreptitious contraceptive use on the part of women was justified by respondents, given unwillingness on the part of some husbands to develop awareness around their family’s needs and the benefits of family planning.

*“There are so many careless husbands. Many husbands don’t know whether their child is going to school or not. But some husbands understand their wives and when wives explain [about FP]-they understand what they should and shouldn’t do.”* (Female, Agra, two or more children)

#### Perceived accuracy and norm consistency

Beyond the propagation of norms, the process, quality, and sources of interpersonal communication that sensitize men and women to contraceptives may affect behavior. In particular, the perceived accuracy of information received by women living in the urban Uttar Pradesh context does not always affect family planning norms uniformly. Irrespective of media or social rhetoric showing family planning in a positive light, adoption of a particular method may be inhibited at the individual level by sharing of negative experiences of family and friends. For example, though many men and women reported condom use as their main method of birth control, myths or negative perceptions continue to limit use. Also, negative experiences of individuals may easily perpetuate norms in a community context, as demonstrated in the following example of IUDs.

*“Sometimes it breaks inside. This had happened with one lady. She used Copper-T, it broke inside and she was badly injured. This information has spread all over the place.”*(Male, Agra, two or more children)

The sharing of perceived risk associated with contraceptive methods is an important but inconsistent way in which norms are communicated. For instance, we see in some cases that despite negative experiences of IUDs and injectables—which led people to discontinue use and create negative perceptions—these methods retained appeal given their effectiveness as a spacing method of clandestine nature. This variability in risk perception may also reflect the knowledge of users; when questioned about the degree to which certain methods were better than others, a woman from Gorakhpur (with only one child) answered “*It depends on if the thing [Copper-T] suits you or not*.” Two consistent norms around method-associated risk appeared in the FGDs: first, the potentially adverse effects that may occur immediately after adopting a female-centric method would decrease over time and, second, that individual responses to methods may differ.

It appears that the primary mechanism through which these norms were disseminated in the community was through interpersonal communication. Regardless of whether certain methods were objectively harmful or risky, their use was governed, to a large extent, by norms perpetuated through interpersonal communication within the community. This is in line with other research [16, 31] showing that discussions among members of a social group often serve to disseminate norms that, in turn, affect social behaviors.

## Discussion

The purpose of this study was to determine the extent to which findings about the relationship between descriptive and injunctive norms, observed in previous studies for other health domains, would also manifest in understanding the use of modern contraceptive methods. We also sought to gain a better understanding about the role of interpersonal communication in normative influences. Quantitative data showed that interpersonal discussions, though low in magnitude, were significantly associated with modern family planning use across all parity groups. Descriptive norms and injunctive norms were associated with modern family planning use for only the high-parity groups. Analyses also revealed that the interaction between norms and interpersonal communication were associated with modern family planning use across all parity groups.

The theory of normative social behavior (TNSB) posits that the influence of descriptive norms on behavior is modified by interpersonal communication and injunctive norms, among others. Support for the first proposition was stronger than that for the moderating role of injunctive norms, but it appears that the TNSB can provide a meaningful lens through which to view contraception-related decision-making. One of the key functions of interpersonal discussion is to transmit information about norms in a community [17]. It is through discussions that individuals come to learn about both the behaviors of others in their social environment and the collective opinions that govern the appropriateness of those behaviors. Thus, it is not surprising that, in our study, interpersonal communication served to boost the influence of descriptive norms on contraceptive behavior.

Qualitative methods adopted in this study enabled us to gain a better understanding about the prevailing norms and patterns of interpersonal communication that perpetuate those norms. Our data suggested that joint families may have a dynamic that differs from nuclear families and that newly married couples may be more influenced by negative prejudices around delayed childbearing and be obliged to have at least one child before seeking family planning services.

Given the influence of interpersonal communication in propagating norms, the accuracy of information disseminated in a community must be of particular concern to public health professionals. Inaccurate information, in our case, negative side effects of certain contraceptives experienced by a minority of users, can be perpetuated in a community and this can take on a life of its own. This social amplification of risk through mass media and other communication networks, for example, is a subject that scholars have long recognized [32]. Our findings add to that literature by recognizing the role of perceived norms in that process. They also point to the need for public health professionals to be cognizant about dominant narratives that exist in a community that may facilitate or hinder intervention goals.

One implication that emerges from this paper pertains to the finding that descriptive norms and injunctive norms surrounding family planning were associated with use of contraceptive methods only among couples with high parity. When couples did not have children or only had one child, their normative beliefs appear not to exercise much impact in their decision to use modern contraceptives. This likely suggests that couples’ desires to have at least two children overpowered normative considerations: it did not matter what the others believed or practiced in this regard. The broader implication of this finding is that norms do not exert their influence uniformly across behaviors and contexts; rather, they are situationally driven.

The interaction effect between interpersonal communication and descriptive norms reinforces the idea that discussions can serve to propagate normative influences in a community. Indeed, unlike laws that are explicitly codified in society, and whose infractions provide well-calibrated sanctions and punishment, norms are socially negotiated, understood, and implemented. Discussions between members of the community serve this social function: it is through discussions that norms derive their meaning.

### Strengths and limitations

The use of cross-sectional data limits our ability to make causal inferences. Although prior studies examining norms have found similar results [25, 33], and our findings are consistent with the theoretical predictions, we cannot rule out alternative explanations for our findings. The use of mixed methods, however, is a strength, which allows us to understand some of the underlying issues in a more in-depth manner. The importance of norms, for example, was readily apparent in the qualitative data, where participants’ perceptions about both descriptive and injunctive norms often shaped their decision-making.

Because for the qualitative data collection we did not conduct couples' interviews — we only interviewed women and men separately—we were unable to gauge the accuracy of self-reported descriptions about interpersonal communication between couples [34]. Likewise, for the quantitative data, we only have women’s reports on interpersonal communication. Hence, we are unable to determine the extent to which reported levels of discussions between couples is corroborated. Nevertheless, this study integrates interpersonal communication with normative influences, a line of inquiry that has not received adequate attention in the literature, despite the fact that norms are inherently social in nature. Thus, while some studies have focused on the importance of inter-spousal communication [35] and others have found that norms are important drivers of contraceptive use [15], this study is unique in delineating the underlying relationships among communication, normative processes, and use of contraceptives.

### Implications for interventions and policy

In contextualizing the interpersonal dynamics between men and women, and their immediate social networks, we see a number of implications for policy and for designing family planning interventions. Our data indicate that attention to parity is critical in developing appropriate programs that are specific to different stages in the reproductive life-course. Newly married couples, for example, appear to be driven by the need to establish their reproductive bona fides by giving birth to their first child soon after marriage. Hence, messages promoting contraceptive use may not resonate with them. Rather, it may be more appropriate to promote prenatal care for this population.

Our dual observations—that interpersonal communication was rather low but that it was a significant predictor of contraceptive use—point to the need to emphasize the importance of and come up with ways to promote interpersonal communication. Further, the substance of communication also needs to be accurate. Marketing campaigns are often built on the creation of a “buzz” around products and services being sold to the public; in the same vein, public health interventions also need to promote interpersonal communication through innovative techniques.

Given India’s tumultuous family planning history [36], current programming and policy requires particular sensitivity to community norms and perspectives. Open interpersonal discussion about family planning is likely to lead to improvements in people’s perceptions about contraception (descriptive norms) and positive support for use (injunctive norms), resulting in more women and couples being able to use modern family planning to meet their current fertility desires. This will lead to improved health and well-being for Uttar Pradesh and beyond.

### Unanswered questions for future research

Findings reported in this paper also point to a number questions that could be raised in future research. It is not known, for example, the extent to which individuals develop normative perceptions based on behaviors they practice. It is possible that those who do not use contraceptive methods conclude that many others are also not doing so; this would provide a strong rationalization for continuing to refrain from use. Using longitudinal studies, future research efforts could better specify that underlying causal mechanism.

Future research could also focus on the nature of the conversations that take place between spouses and others who are influential in women’s decisions to use contraceptive methods. We do not know, for example, the extent to which women receive consistent information from various sources, including their spouses, providers, friends, and others. One can imagine that consistently receiving pro-contraceptive use information from all sources would be much more influential than contradictory information from the various parties. How women make sense of contradictory information and whose information they weight more heavily are also issues that have not received adequate attention in the literature. Exploring these in varied contexts would allow for us to elaborate upon on transferability of the situationally driven normative influences presented in our study.

## Footnotes

1 Ethics approval was granted by University of North Carolina (Speizer, Principal Investigator) for the quantitative data and the Johns Hopkins Bloomberg School of Public Health (Rimal, Principal Investigator) for the qualitative data (work was done during Rimal’s tenure at Johns Hopkins University).
